# Hepatic vein resection and reconstruction for liver malignancies: expanding indication and enhancing parenchyma-sparing hepatectomy

**DOI:** 10.1093/bjsopen/zrab121

**Published:** 2022-01-12

**Authors:** Fumihiro Kawano, Yoshihiro Mise, Junji Yamamoto, Atsushi Oba, Yoshihiro Ono, Takafumi Sato, Yosuke Inoue, Hiromichi Ito, Yu Takahashi, Akio Saiura

**Affiliations:** 1 Department of Hepatobiliary and Pancreatic Surgery, Cancer Institute Hospital, Japanese Foundation for Cancer Research, Ariake, Tokyo, Japan; 2 Department of Hepatobiliary–Pancreatic Surgery, Juntendo University School of Medicine, Hongo, Tokyo, Japan

## Abstract

The short-term outcomes after hepatic vein reconstruction (HVR) were evaluated in 41 patients. HVR can be performed safely using autologous vein graft. HVR can increase the functional remnant liver volume, facilitating parenchyma-sparing one-step hepatectomy as well as expanding resectability even in patients with all major hepatic veins involved.

*Dear Editor*

Hepatectomy with hepatic vein (HV) resection and hepatic vein reconstruction (HVR) has been performed previously to expand resectability for patients with otherwise unresectable liver tumours. Owing to recent technical advances in liver surgery, HVR following minor hepatectomy has also been proposed as an alternative to major hepatectomy to spare uninvolved liver parenchyma^1^. This letter reports the feasibility of HVR.

Patients who underwent hepatectomy with HVR for liver malignancy from 2002 to 2018 at The Cancer Institute Hospital of Japan were reviewed retrospectively and postoperative short-term outcomes were analysed. Patients were categorized in two groups according to the indications for HVR: a definitive HVR (D-HVR) group in which liver tumours were unresectable without HVR or staged hepatectomy, and a parenchyma-sparing HVR (P-HVR) group in which HVR was applied to avoid extended major hepatectomy (*Fig. 1*).

**Fig. 1:**
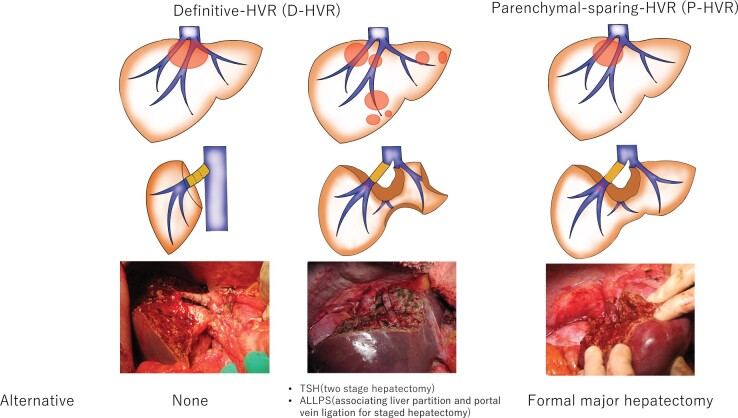
Hepatic vein reconstruction categorized in two groups according to indication HVR, hepatic vein reconstruction; TSH; ALLPS.

Resectability was evaluated with contrast-enhanced CT with or without supplemental use of MRI or PET^2^. Safety criteria for liver resection were described previously as follows: patients should not have co-morbid conditions that preclude major hepatic resections, and all liver lesions should be amenable to resection with clear margins, leaving at least 30 per cent of remnant liver without potentially ischaemic or congested areas in patients with normal liver function. Liver function was assessed by indocyanine green retention rate at 15 minutes (normal, less than 10 per cent). Venous congestion following sacrifice of the involved hepatic vein was evaluated using three-dimensional image-processing software prior to surgery^3^.

During the study period, 2907 hepatectomies were performed and 41 patients (1.4 per cent) underwent 43 HVRs. D-HVR and P-HVR were performed in 23 patients (56 per cent) and in 18 patients (44 per cent) respectively. Patient demographics and surgical outcomes are summarized in *Table S1*. A total of 28 patients underwent segmental HV resection. In six patients (21 per cent) the HV was reconstructed with end-to-end direct anastomosis and in 23 patients (79 per cent) by interposing autograft. Customized saphenous vein graft was the most frequently used interposition graft (20 patients)^4^. External iliac vein graft and left portal vein extracted from the resected specimen were used in two and one patient, respectively. Wedge resection alone was performed in 13 patients. One patient had wedge resection in addition to segmental HV resection. All wedge resections were repaired using an autologous patch. The opened umbilical vein was most frequently used (11 procedures). In addition, left hepatic vein (1 patient), left portal vein (1 patient) and ovarian vein (1 patient) were used for patch repair after wedge HV resection. In the D-HVR group, all three HVs were resected in 12 patients.

Major complications, defined by Clavien–Dindo classification grade 3 or above, occurred in four patients of the D-HVR group (17 per cent)^5^. No patient had major complications in the P-HVR group. The 90-day mortality rate was zero in both groups. The resected weight of the liver was significantly greater in the D-HVR group compared with the P-HVR group (*P* < 0.001). One- and 3-year graft-patency rates were 96 and 77 per cent in the segmental resection group, and 91 and 91 per cent in the wedge resection group respectively.

This study reports a large series of HVR characterized by the exclusive use of autologous grafts avoiding artificial or cryopreserved grafts that can be not easily available in clinical practice. In addition, the limited use of advanced procedures such as total hepatic vascular exclusion and extracorporeal temporal bypass might have contributed to favourable short-term outcomes with no deaths, even though all major hepatic veins were resected in 12 patients (27 per cent). Given these promising results, such an approach might not only expand the indications for surgery for unresectable liver malignancies, but also broaden the indications for parenchyma-sparing resections avoiding a two-step or major hepatectomy.

*Disclosure*. The authors declare no conflicts of interest.

## Supplementary material

Supplementary material is available at *BJS Open* online.

## Supplementary Material

zrab121_Supplementary_Data
