# Supplementary material for: Hepatic vein resection and reconstruction for liver malignancies: expanding indication and enhancing parenchyma-sparing hepatectomy
Source: BJS Open. 2022 Jan 12;5(6):zrab121. doi: 10.1093/bjsopen/zrab121 (PMC8759503; doi:10.1093/bjsopen/zrab121)
Supplement: Supplementary file 1 [file zrab121_supplementary_data.docx]

Supplementary Table 1 .Pre-, intra- and postoperative profile of all patients who underwent HVR according to indication for HVR

			D-HVR (n=23)	*P-HVR (n=18)*	
Age			60 (36-77)	59 (45-74)	0.675
Sex (male)			16 (69%)	7 (38)	0.064
Preoperative Chemotherapy			15 (65%)	10	0.748
Type of Tumor					0.243
	Liver metastases		20	18	
	ICC		3	0	
ICG R 15 <10%＊			9 (39%)	9 (50%)	0.057
Maximun size			5 (1.7-9)	2.7 (1.2-4.9)	<0.0001
tumor number			7 (1-36)	2 (1-21)	0.037
Number of involved HV					<0.0001
	1		3	12	
	2		8	6	
	3		12	0	
Type of Hepatectomy					0.038
	minor hepatectomy		13	16	
	Major hepatectomy		10	2	
Type of HVR					0.067
	Segmental		18	9	
	Patch		4	9	
	Segmental + Patch		1	0	
TSH/ALLPS					
	TSH		1	0	
	ALLPS		1	0	0.19
Estimated blood loss (ml)			1550 (580-2600)	755 (220-3700)	0.037
Operating time (min)			565 (355-935)	437.5 (196-985)	0.091
Resected weight (g)			437 (128-774)	149 (68-510)	<0.001
Major morbidity			4 (17.3%)	0 (0%)	0.254
	Bile leakage ¶		2 （GradeB)	0	0.200
	Posthepatectomy liver failure ¶		2 (GradeB)	0	0.189
	Bleeding		0	0	N.A
	PVE,PE		0	0	N.A
	Ascitis		4	2	0.572
	Pleural effusion		3	2	0.851
	Surgical site infection		5	2	0.572
90-day mortality			0	0	
R0			12 (52.1%)	10 (55.5%)	1
Hospital stay			19 (10-71)	14 (11-26)	0.008

Data are median (range) or number (%)

¶ The International Study Group of Liver Surgery (ISGLS)

＊NA(n=7)
